# Non-nutritive sweeteners possess a bacteriostatic effect and alter gut microbiota in mice

**DOI:** 10.1371/journal.pone.0199080

**Published:** 2018-07-05

**Authors:** Qiao-Ping Wang, Duncan Browman, Herbert Herzog, G. Gregory Neely

**Affiliations:** 1 University of Sydney, Dr. John and Anne Chong Lab for Functional Genomics, Charles Perkins Centre, Camperdown, NSW, Australia; 2 University of Sydney, School of Life and Environmental Sciences, Camperdown, NSW, Australia; 3 School of Pharmaceutical Sciences (Shenzhen), Sun Yat-sen University, Guangzhou, China; 4 Neuroscience Division, Garvan Institute of Medical Research, Darlinghurst, Sydney, NSW, Australia; Universite Paris-Sud, FRANCE

## Abstract

Non-nutritive sweeteners (NNSs) are widely used in various food products and soft drinks. There is growing evidence that NNSs contribute to metabolic dysfunction and can affect body weight, glucose tolerance, appetite, and taste sensitivity. Several NNSs have also been shown to have major impacts on bacterial growth both *in vitro* and *in vivo*. Here we studied the effects of various NNSs on the growth of the intestinal bacterium, *E*. *coli*, as well as the gut bacterial phyla Bacteroidetes and Firmicutes, the balance between which is associated with gut health. We found that the synthetic sweeteners acesulfame potassium, saccharin and sucralose all exerted strong bacteriostatic effects. We found that rebaudioside A, the active ingredient in the natural NNS stevia, also had similar bacteriostatic properties, and the bacteriostatic effects of NNSs varied among different *Escherichia coli* strains. In mice fed a chow diet, sucralose increased Firmicutes, and we observed a synergistic effect on Firmicutes when sucralose was provided in the context of a high-fat diet. In summary, our data show that NNSs have direct bacteriostatic effects and can change the intestinal microbiota *in vivo*.

## Introduction

Sweet-tasting food is preferred by most people and preloading experiments show that sweet taste, whether delivered by sugar or artificial sweeteners, enhances human appetite [1]. Because of the concern that high sugar intake can increase the risk of developing obesity, pre-diabetes, type 2 diabetes, and cardiovascular disease, non-nutritive sweeteners (NNSs) are increasingly used to replace sugars. NNSs are marketed as dieting tools, and consumers may intuitively choose NNSs over sugar to maintain or lose weight. There is emerging evidence indicating that NNSs have unanticipated effects on human health. NNS consumption has been associated with increased weight gain in rodents [2–6] and humans [6, 7], and glucose intolerance in rodents and humans [6, 8, 9], as well as increased appetite and altered taste sensitivity in *Drosophila* [10, 11].

Commercially available NNSs include sucralose, acesulfame potassium (Ace K), saccharin, aspartame and stevia. Sucralose, one of the most commonly consumed synthetic NNSs, exists in over 4500 products and accounts for 62% of the $1.146 billion global artificial sweetener market. More recently, naturally occurring NNSs like stevia has been introduced into the market [12]. Steviol glycosides are extracted from the plant *stevia Rebaudiana* and are perceived as 200–300 times sweeter than sucrose [13, 14]. Commercial steviol glycoside mixtures contain two main active components: stevioside (10–70%) and rebaudioside A (20–70%).

Both synthetic and natural NNSs have been shown to exert bacteriostatic effects on a variety of bacteria species. Ace K, cyclamate and saccharin inhibit the anaerobic fermentation of glucose by rat gut flora [15]. Sucralose, saccharin and aspartame inhibit the growth of two common periodontal pathogenic bacterial species [16]. Sucralose also inhibits the growth of the oral bacterium *Streptococcus spp*. [17, 18] and various environmental microbes [19, 20]. Stevia extract exerts bactericidal effects on *E*. *coli* O157:H7, but not *Bifidobacterium* and *Lactobacillus* [21], whereas stevia glycosides inhibit the growth of *Lactobacillus reuteri* in a strain-specific manner [22]. Importantly, many NNSs are not efficiently absorbed through the intestine and may build up in the lumen of the gut.

The gut microbiota consists of hundreds of bacterial species and is involved in multiple physiological functions such as carbohydrate and amino acid metabolism [23]. Dysfunction of gut microbiota is associated with obesity and insulin resistance [24]. For example, high-fat diets have been shown to cause an increase in phylum Firmicutes and a decrease in Bacteroidetes, and these changes are sufficient to promote metabolic dysfunction [25, 26]. There is growing evidence indicating that NNSs also have an important impact on gut microbiota in rodents. Saccharin (7.5%, w/v) increases gut aerobic bacteria and decreases anaerobic bacteria in rats [27], and sucralose (1.1–11 mg/kg/d) alters gut microflora in Sprague-Dawley rats [28]. Aspartame (5–7 mg/kg/d) induces significant alterations in intestinal bacteria composition with increased abundance of gut Enterobacteriaceae and *Clostridium letptum* in Sprague-Dawley rats [29]. Furthermore, the NNSs, saccharin (0.5%, w/v), aspartame (0.4%, w/v), and sucralose (0.5%, w/v) cause dysbiosis of gut bacteria in C57BL/6 mice [8]. Sucralose (15 mg/kg/d) but not Ace K (15 mg/kg/d) affects the amount of gut *Clostridium cluster XIVa* in C57BL/6 mice [30]. Ace K (37.5 mg/kg/d) alters gut microbiota composition in CD-1 mice [31]. Importantly, all studies on NNS-microbiota interactions so far have been carried out in adult animals, while the effect of NNSs on gut microbiota in young animals remains unexplored.

In order to investigate the effects of NNSs on the gut microbiome at the organismal level, we performed phylogenetic analysis using next generation sequencing on the faeces of adolescent mice fed with NNSs. Furthermore, to dissect the effects of NNSs on commensal bacterial species, we use an *in vitro E*. *coli* bacterial growth assay. Our study demonstrates that NNSs possess a bacteriostatic effect and alter gut microbiota in mice.

## Materials and methods

### Bacterial strains

*Escherichia coli* strain HB101 and K-12 were purchased from Promega (US) and were maintained in Luria Bertani’s Broth (LB).

### Non-nutritive sweeteners

Sucralose, saccharin, acesulfame potassium and rebaudioside A were purchased from Sigma. LB media and agar were obtained from Invitrogen.

### Liquid culture assay

Sucralose, saccharin, acesulfame potassium and rebaudioside A or an equal molarity of sodium chloride or sucrose were dissolved in Luria’s Broth (LB). *E*. *coli* HB101 and *E*. *coli* K-12 strains were cultured to mid-log growth phase in LB and inoculated using 30 μL of culture in 3 mL of culture medium (1:100) and incubated at 37°C at 220 rpm in a shaking incubator. After 5 hours, the cultures were removed from the incubator, placed in disposable, plastic cuvettes and the optical density determined by spectrophotometry at 600 nm (OD_600_). LB without NNSs were used as background references and were generally found to be within 0.006 OD_600_ units of the water blank.

### LB agar plate assay

For rebaudioside A experiments, 10-fold serial dilutions of rebaudioside A-treated cultures were performed in LB and a volume of 200 μL of diluted cultures were spread on LB-agar plates and incubated at 37°C. After 24 hours, colonies were counted manually on plates with moderate densities (generally the plates representing 10^−6^ to 10^−7^ of the initial culture concentrations) and colony forming units (CFUs) per millilitre of culture was determined. For solid media sucralose experiments, sucralose was dissolved in molten LB-agar and used to make sucralose plates with 1.25% (w/v) sucralose and 2.5% (w/v) sucralose. Colony area is the area in square millimetres that is occupied by each bacterial colony. This was done by calculating the pixel to area ratio in Image J. Bacterial cultures were grown to mid-log growth phase, serially diluted with 10-fold dilutions, plated on treated LB-agar plates using 200 μL of diluted culture and placed in an incubator at 37°C. Standard LB-agar plates were used as treatment controls. After 24 hours, the plates were photographed. Colonies were counted and colony area measurements were determined using Image J software (https://imagej.nih.gov/ij/).

### Mouse studies

Experimental procedures were performed under the ethical standards approved by the Garvan Institute of Medical Research Animal Care and Use Committee. Chow diet (8% calories from fat, 21% calories from protein, 71% calories from carbohydrate, 2.6 kilocalorie (kcal/g)) and high fat diet (HFD, 23% calories from fat, 19.4% calories from protein, 48.2% calories from carbohydrate, 4.7% calories from crude fibre, 4.7% calories from acid detergent fibre, 4.78 kcal/g) were purchased from Gordon’s Specialty Feeds, Glen Forrest, WA, Australia. C57BL/6 mice were housed in pairs in a 12 h light/dark cycle. Animals at 5 weeks of age were divided into four dietary groups based on body weight; chow (12% kcal fat) + water, chow + sucralose solution, high fat diet + water (HFD, 60% kcal fat), HFD + sucralose solution for 8 weeks (n = 8 mice per group). Sucralose (Sigma) solution was made by directly adding sucralose to drinking water (2.5%, w/v). All animals had access to food and fluid ad libitum for an additional 8 weeks prior to sacrifice. The faeces were collected at Zeitgeber time 3 in the daylight cycle (week 5, 8 and 12) for gut microbiota analysis.

### Bacterial 16s RNA sequencing and analysis

Faeces were collected at week 5, 8 and 12 in both chow and HFD fed mice. Total bacterial DNA was extracted and 16s rDNA were sequenced and mapped according to taxonomy. Mice faecal bacterial DNA were extracted by uBiome kit and 16s rDNA were amplified and sequenced by Illumina NextSeq 500. The sequences were mapped according to NCBI taxonomy. The gut microbiota abundance was expressed as percentage of the total amount. For *α*-diversity, a Shannon index was initially calculated in the vegan package [32] in R. The original sequence data is available at SRA accession: SRP148650.

### Statistical methods

One-way analysis of variance (ANOVA) with Bonferroni’s test for multiple comparisons was used for *in vitro* sucralose study. Wilcoxon match-pairs signed rank test was used for rebaudioside A *in vitro* study. Two-way analysis of variance (ANOVA) with Bonferroni’s test for multiple comparisons was used for *in vivo* mouse study.

## Results

### Sucralose possesses a bacteriostatic effect on *E*. *coli in vitro*

**Fig 1:**
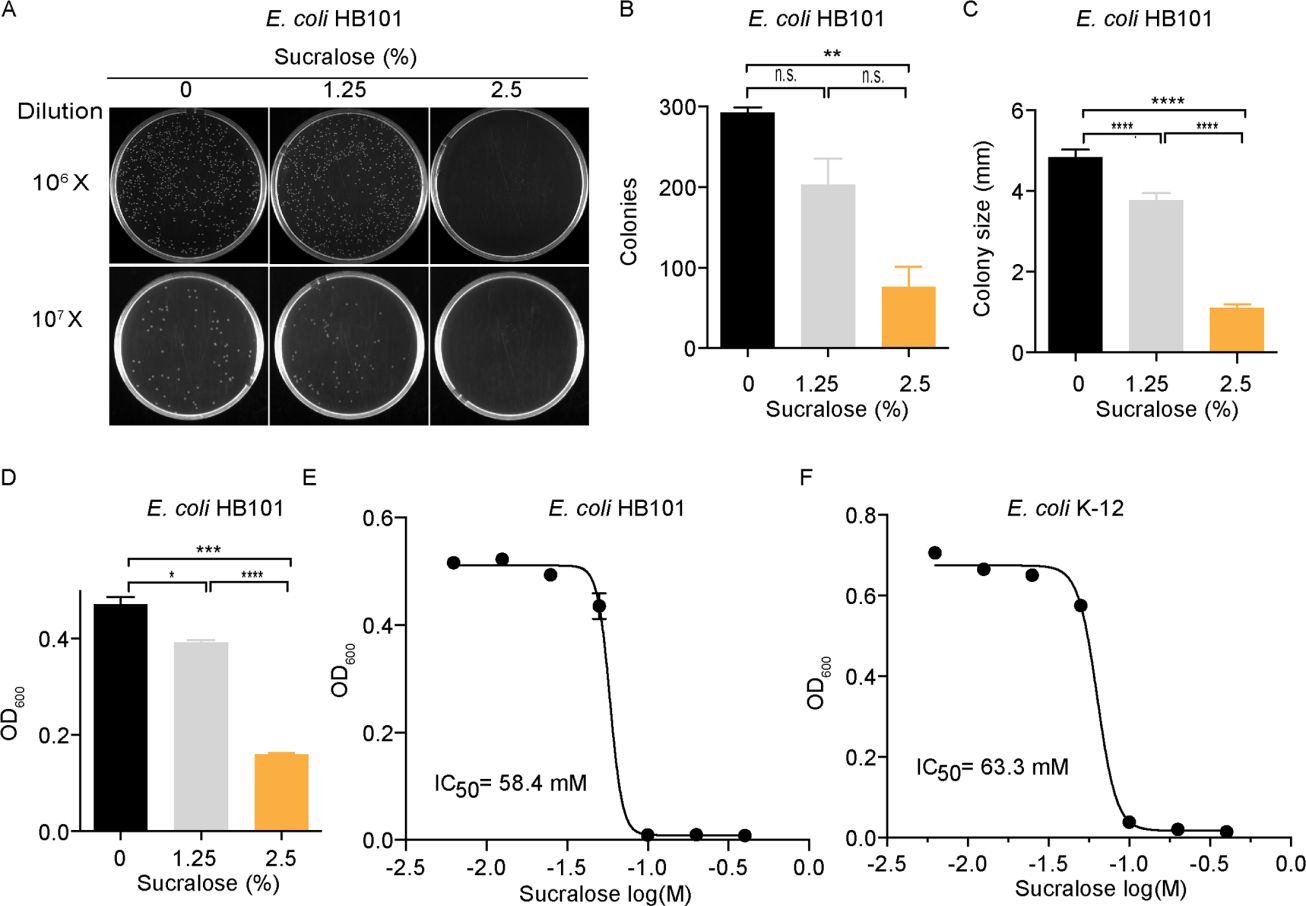
Sucralose possess bacteriostatic effect on *E*. *coli in vitro*. (A) Sucralose inhibited *E*. *coli* HB101 growth on agar plates. *E*. *coli* culture in the exponential growth phase was diluted to 10^−7^ or 10^−6^ /ml and inoculated on LB-agar plates containing 0%, 1.25% and 2.5% sucralose (w/v). Colonies were counted after 24h. (B) Quantification of colonies, n = 3 replicated plates. (C) Colony size was also reduced on sucralose-containing plates, n = 45–52 colonies. (D) Sucralose inhibits *E*. *coli* HB101 growth in liquid culture, n = 3. (E-F) The IC_50_ of sucralose on the growth of (E) the *E*. *coli* HB101 strain and (F) the *E*. *coli* K-12 strain, n = 3. All data represent mean ± S.E.M. One-way analysis of variance (ANOVA) with Bonferroni’s test was used for multiple comparisons in Fig 1B–1D. *p<0.05, **p <0.01, ***p <0.001, ****p <0.0001. n.s.,not significant.

We first assessed the effect of sucralose on the growth of the *E*. *coli* HB101 strain in both solid media and in liquid culture. Sucralose significantly reduced the number of *E*. *coli* colonies grown on impregnated LB-agar plates in a dose-dependent manner (Fig 1A): 30% fewer colonies were observed on plates containing 1.25% sucralose/LB-agar (w/v), whereas 74% fewer colonies were observed on plates with 2.5% sucralose/LB-agar (w/v) (Fig 1B). Furthermore, sucralose reduced the mean size of bacterial colonies by 22% and 77% in plates treated with 1.25% and 2.5% sucralose, respectively (Fig 1C). Similarly, in liquid culture, the growth of *E*. *coli* HB101 bacteria was inhibited by 17% and 66% in liquid, LB medium containing 1.25% and 2.5% sucralose (w/v), respectively (Fig 1D). The IC_50_ of sucralose on the growth of *E*. *coli* strains HB101 and K-12 were determined. *E*. *coli* HB101 was slightly more sensitive (IC_50_ = 58.4 mM; Fig 1E) to sucralose when compared to the *E*. *coli* K-12 strain (IC_50_ = 63.3 mM; Fig 1F). Thus, sucralose can exert a bacteriostatic effect on *E*. *coli* bacteria *in vitro*.

### Ace K and saccharin possess a bacteriostatic effect on *E*. *coli in vitro*

**Fig 2:**
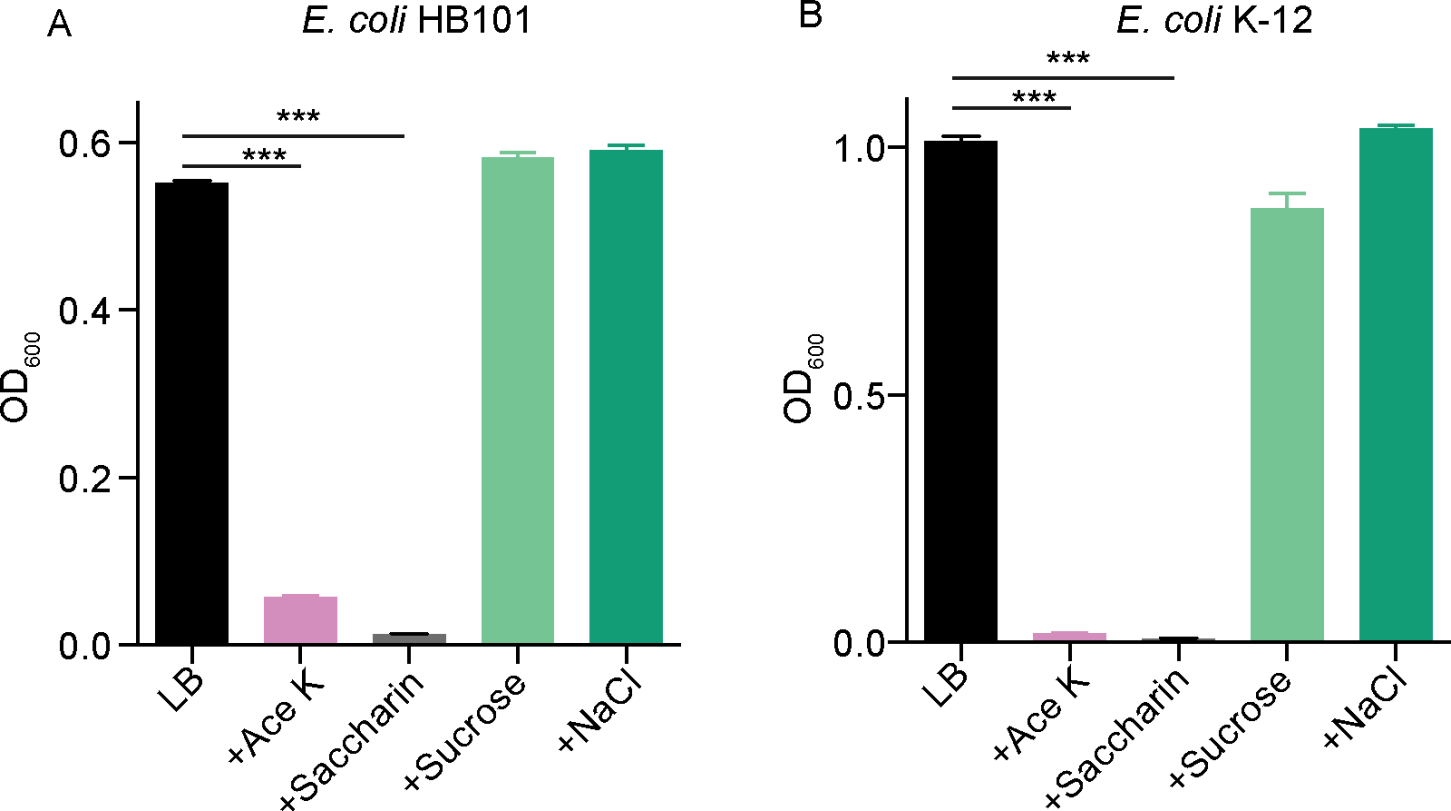
Ace K and saccharin show bacteriostatic effects on *E*. *coli in vitro*. Ace K (2.5%, w/v) and saccharin (2.5%, w/v) inhibited (A) *E*. *coli* HB101 and (B) *E*. *coli* K-12 strains in LB liquid culture, n = 3. All data represent mean ± S.E.M., One-way analysis of variance (ANOVA) with Bonferroni’s test was used for multiple comparisons. ***p <0.001.

To investigate whether a bacteriostatic effect on enteric bacteria was a general feature of synthetic NNSs, we expanded our studies to test the effects of Ace K and saccharin on the growth of *E*. *coli*. Both Ace K and saccharin possessed a strong inhibitory effect on the growth of *E*. *coli* strains (Fig 2A and 2B), similar to that of sucralose. Conversely, iso-osmolar concentrations of neither sucrose nor NaCl, dissolved in LB significantly affected bacterial growth (Fig 2A and 2B). Ace K (2.5% w/v) inhibited the growth of *E*. *coli* HB101 by 90% (Fig 2A) and *E*. *coli* K-12 by 98% (Fig 2B). Saccharin (2.5% w/v) also inhibited the growth of *E*. *coli* HB101 by 98% (Fig 2A) and *E*. *coli* K-12 by 99.5% (Fig 2B), respectively. Thus, the ability to selectively inhibit the growth of enteric bacterial species is a common property of artificial NNSs, including Ace K and saccharin.

### The natural NNS stevia exerts a bacteriostatic effect on enteric bacterial species *in vitro*

**Fig 3:**
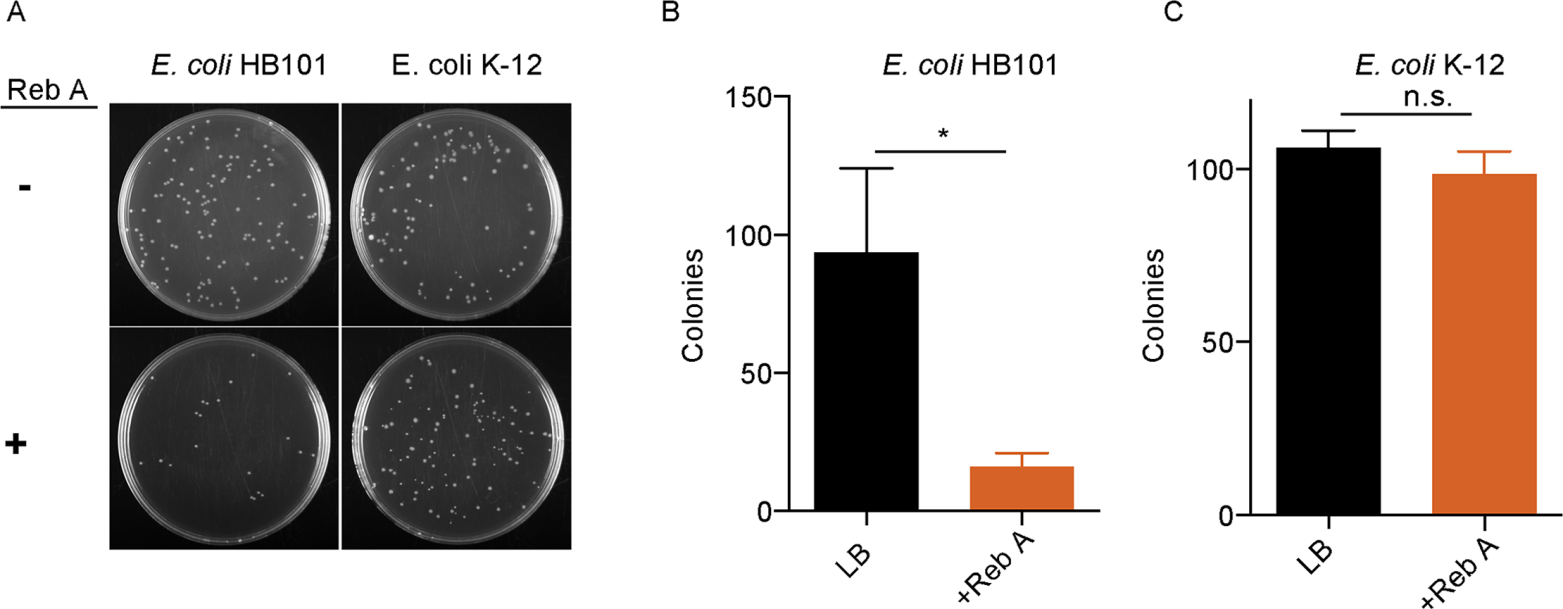
The natural NNS reb A possesses a selective bacteriostatic effect on *E*. *coli in vitro*. Reb A (2.5%, w/v) inhibited the growth of *E*. *coli* HB101 colonies (A, B), but not *E*. *coli* K-12 colonies (A, C), n = 3. All data represent mean ± S.E.M., Wilcoxon match-pairs signed rank test was used. * p<0.05. n.s., not significant.

While artificial sweeteners had an impact on bacterial growth, we were interested in determining if naturally-occurring NNSs would have the same bacteriostatic effect on *E*. *coli* growth. Since rebaudioside A eventually formed a precipitate in liquid cultures, we assessed growth by a colony-forming assay instead of by optical density measurements of liquid cultures. Similar to synthetic sweeteners, Reb A significantly impeded *E*. *coli* HB101 growth (Fig 3A). The number of *E*. *coli* HB101 colonies were reduced by 83% on plates spread with liquid cultures containing 2.5% reb A (w/v) compared to control plates spread with *E*. *coli* cultured in LB alone (Fig 3B). Interestingly, reb A did not impact the number of *E*. *coli* K-12 colonies (Fig 3A and 3C). Thus, similar to synthetic sweeteners, the natural NNS, rebaudioside A, exerts a selective bacteriostatic effect on host gut flora species.

### Sucralose consumption affects bodyweight and promotes faecal output in mice

**Fig 4:**
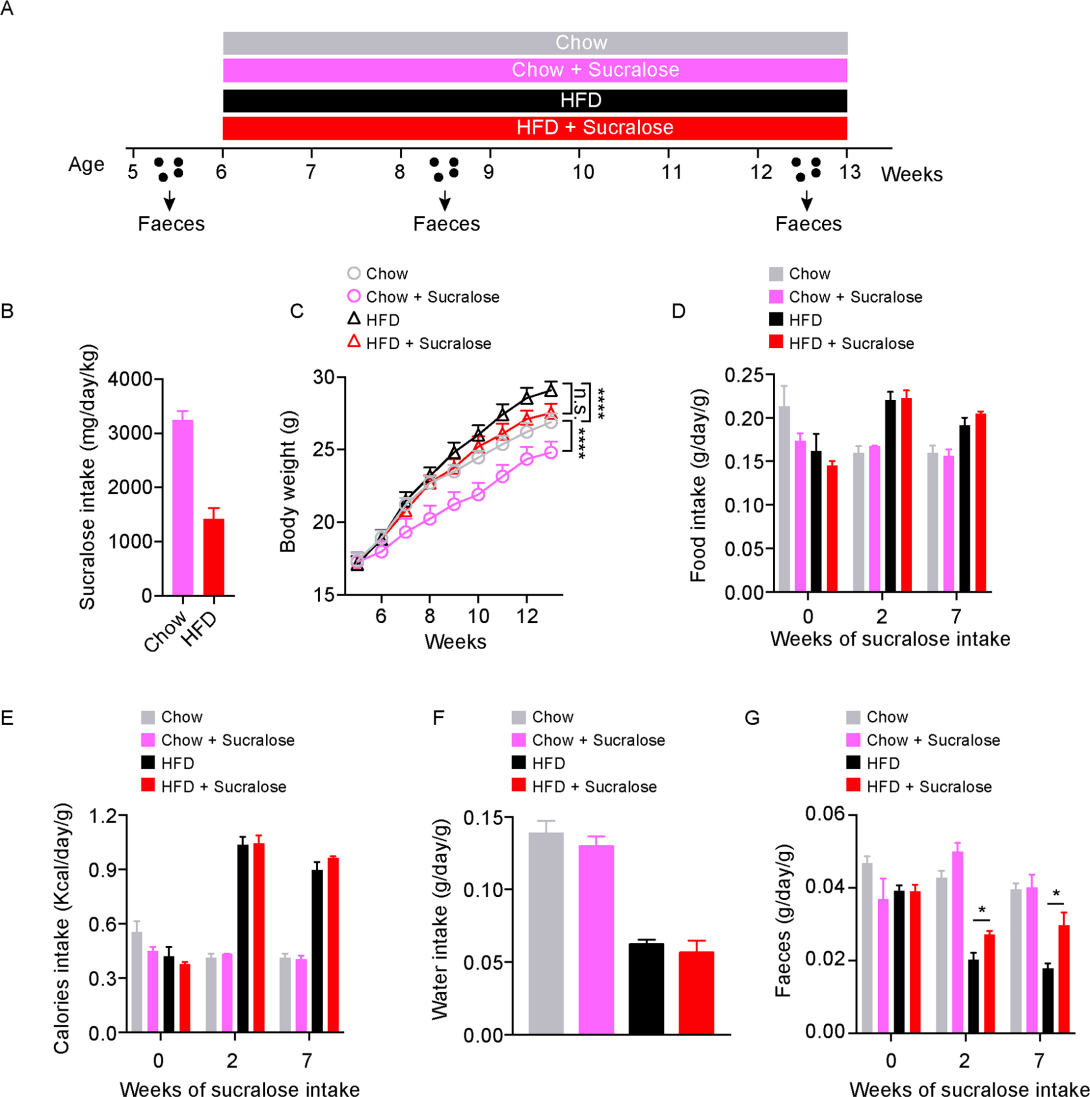
Sucralose promotes faecal excretion in mice. (A) Schematic for the mouse study. (B) Sucralose intake. (C) Body weight. (D) Food intake. (E) Calories intake. (F) Water intake. (G) Faecal weight. All data n = 8 mice per group, represented as mean ± S.E.M., two-way analysis of variance (ANOVA) with Bonferroni’s test for multiple comparisons was used. *p<0.05, ****p <0.0001, n.s., not significant.

We next tested the effects of NNSs *in vivo*. Young (5-week-old) mice were fed normal chow or a high-fat diet (HFD) in the presence or absence of a 2.5% (w/v) sucralose solution for 8 weeks (Fig 4A), the sucralose dose was based on the IC_50_ of *E*. *coli* K-12 *in vitro* (see Fig 1F). Based on water consumption, sucralose intake was ~3.3 mg/kg/d bodyweight in the normal chow group, and ~1.5 mg/kg/d in the HFD group (Fig 4B), which is roughly 300 to 600 times higher than recommend average daily intake (5 mg/kg/d) for humans. As expected, mice gained significantly more body weight with HFD *versus* normal chow (F_8,56_ = 8.531, P<0.0001); conversely, sucralose significantly reduced the body weight of mice fed with normal chow (F_8,56_ = 7.217, P<0.0001), but not with HFD (F_8,56_ = 1.675, P = 0.1250) (Fig 4C). There were no significant differences in food intake (Fig 4D), calorie intake (Fig 4E) or water intake (Fig 4F) between control and sucralose groups in mice fed with normal chow or HFD. However, sucralose significantly increased faecal output in both normal chow or HFD-fed mice (Fig 4G).

### Sucralose alters gut microbiota in young mice

**Fig 5:**
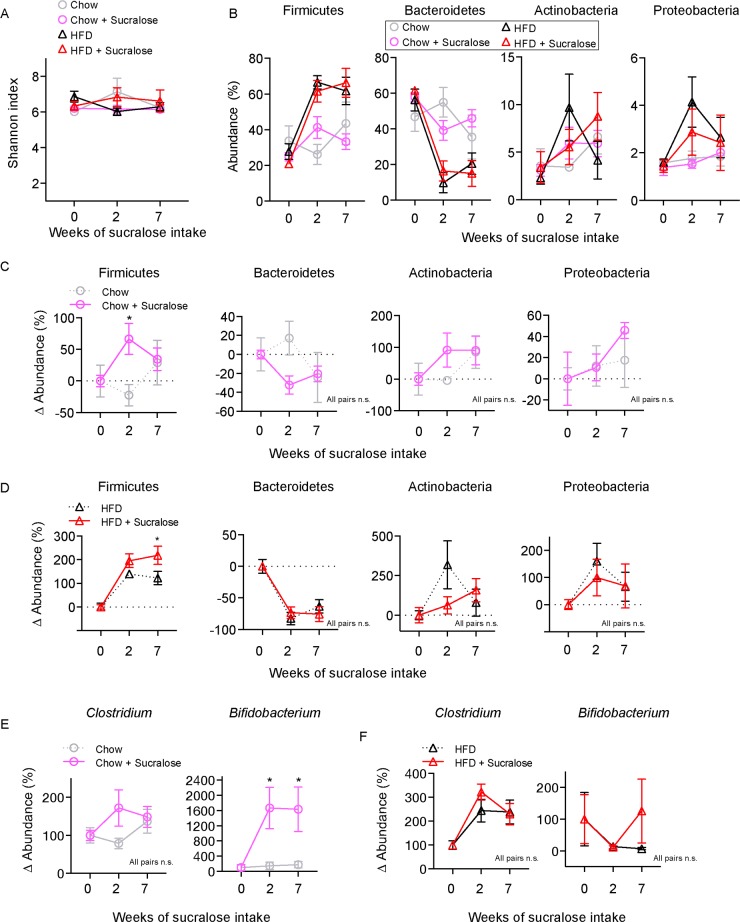
The NNS sucralose alters gut microbiota in mice. (A) *Alpha* diversity of gut microbiota for the diets used. (B) The abundances of the major phyla of gut microbiota after various diets. (C) Relative changes in abundance of major microbiota phyla in chow fed mice. (D) Relative changes in abundance of major microbiota phyla in HFD fed mice. (E) Relative changes in genus of *Clostridium* and *Bifidobacterium* in chow fed mice. (F) Relative changes in genus of *Clostridium* and *Bifidobacterium* in HFD fed mice. All data n = 8 mice per group represented mean ± S.E.M., Two-way analysis of variance (ANOVA) with Bonferroni’s test for multiple comparisons was used. * p <0.05, n.s., not significant.

We collected faeces for bacterial 16s rDNA sequencing to determine the impact of sucralose-containing diets on gut microbiota. There was no significant difference in *alpha* diversity between control and sucralose in normal chow or HFD-fed mice (Fig 5A). 16s rDNA quantification was performed for each condition and mean % abundance of major microbiota populations was evaluated (Fig 5B). Since baseline microbiota composition varied between individuals, we generated relative abundance values (Δ abundance) for each animal and present mean change in abundance (Fig 5C–5F). When compared to chow-only mice, sucralose-exposed chow mice exhibited a significant increase (p<0.05) in Δ abundance for Firmicutes and a trend showing decrease in Bacteroidetes (p = 0.117), however no change in Actinobacteria and Proteobacteria was observed (Fig 5C). In the context of HFD, sucralose-exposed mice again show a significant and long-lasting increase in Firmicutes when compared to HFD controls (p<0.05), while Bacteroidetes species were reduced in both groups (Fig 5D). At the genus level, sucralose did not affect *Clostridium*, however *Bifidobacterium* was significantly (p<0.05) increased in the context of chow (Fig 5E) but not HFD (Fig 5F). Together, these data show that, similar to HFD, sucralose can impact the microbiota composition, and these effects are additive.

## Discussion

NNSs have been associated with metabolic dysfunction but the underlying mechanisms remain unclear. Recent studies reveal that NNSs affect metabolic traits through gut microbiota [8] or an imbalance in energy intake and taste perception [10]. In this study, we investigated the direct effect of NNSs on *E*. *coli* bacteria *in vitro* and on gut microbiota *in vivo*. Our results showed that NNSs exert strong bacteriostatic effects on bacteria *in vitro*, which is consistent with previous studies [15, 17–20, 27]. Moreover, we showed that *in vivo*, sucralose alters gut microbiota promoting an increase in Firmicutes, and the effects of sucralose synergized with HFD to further augment Firmicutes.

Although there are a variety of compounds used as NNSs, all have the ability to mimic the taste of sugar in some regard. So far, all NNSs we have tested show a bacteriostatic effect in some bacterial species. However, the effect on different bacteria varies, even within species [17]. In this study, *E*. *coli* K-12 was more sensitive than *E*. *coli* HB101 to Ace K and sucralose, while *E*. *coli* HB10 was more sensitive to stevia. This difference implies that divergent mechanisms are responsible for the observed bacteriostatic effects. There is some evidence pointing to a mechanism whereby NNSs exert bacteriostatic effects through inhibition of metabolic enzymes or by altering nutrient transportation, or processes that are essential for growth [19, 27]. This difference in susceptibility may in part explain the alterations in bacterial abundance we observed *in vivo*. Given that there are hundreds of bacteria species living in our intestine[33], NNSs may selectively inhibit the survival of some bacterial populations thus causing a change in the balance of the overall gut microbiota. Of note, our finding that sucralose alters gut microbiota may be a general mechanism for all NNSs, many of which are not efficiently absorbed and may become concentrated in the intestine. Of note, there is some evidence that aspartame can also alter the intestinal microbiota in rats [29], however the mechanism for this effect is unclear, as aspartame is rapidly hydrolysed in the upper GI tract and would not likely directly interact with microbiota in the lower intestine.

The abundance and diversity of bacterial species comprising the gut microbiota are in dynamic flux; however, the phyla Bacteroidetes, Firmicutes, Actinobacteria and Proteobacteria seem to dominate in intestine. Previous studies showed that Bacteroidetes is reduced and Firmicutes is increased in obese humans [33–35] and that sucralose can cause dysbiosis of gut bacteria in rat [28] and metabolic dysregulation in mice [8]. Our results here highlight that sucralose promotes an increase in Firmicutes, which is partially consistent with the microbiota alterations reported in similar studies with aspartame or Ace K [8, 29, 30]. Of note, in this study, dietary fibre content was different between chow and HFD and may represent a confounding variable, however HFD promotes significant weight gain, and increases Firmicutes while decrease Bacteroidetes abundance in the gut, which is consistent with previous results for HFD [25, 26]. Although HFD caused a significant increase in *Clostridium* and reduction in *Bifidobacterium*, sucralose did not have additional effects on these bacteria.

The effects of NNSs on weight gain are controversial. Some studies have associated NNS consumption with weight gain in humans, while other studies did not report these effects [36–40]. In our study, sucralose did not promote weight gain either in the context of chow or HFD, and with young mice, we actually observed a decrease in body weight in response to high dose sucralose. This may be due to the high dose of sucralose used in the study, which could be considered super-physiological. Moreover, we found that sucralose promoted increased faecal output, regardless of diet, which could impede nutrient absorption and promote weight loss. Alternatively, it is possible that this study design was not long enough to see increased weight gain, as long-term weight gain and gain in waist circumference are observed in adult humans who consume NNSs daily [6, 41]. Interestingly, maternal consumption of NNSs also appears to impact weight gain in infants and children [41, 42]. Moreover, routine consumption of NNS is associated with increased risk of cardiometabolic diseases [43].

Together our data show that a variety of NNSs can have a direct effect on commensal bacteria. This study further reinforces the notion that NNSs are not biologically inert; rather, consumption of NNSs alters the relative proportion of intestinal microbial phyla through a selective bacteriostatic effect. The mechanisms for this activity and relevance to human disease warrant further investigation.

## Supporting information

S1 Table Gut microbiota abundance. The raw sequence reads of gut microbiota. Group: week 0, 2 and 7 indicates weeks of sucralose consumption; Count, read count; Tax-name: taxonomy name; Tax-rank, ranking of the taxonomic classification, Percentage: relative abundance normalized to all counts in each sample.
(XLSX)
